# ‘It’s basically ‘have that or die’’: a qualitative study of older patients’ choices between dialysis and conservative kidney management

**DOI:** 10.1136/bmjopen-2024-095185

**Published:** 2025-03-07

**Authors:** Barnaby Hole, Leila Rooshenas, Rachael Morton, Fergus Caskey, Miranda Scanlon, Joanna Coast, Lucy Selman

**Affiliations:** 1 Richard Bright Renal Unit, North Bristol NHS Trust, Bristol, UK; 2 Population Health Sciences, University of Bristol, Bristol, UK; 3 The University of Sydney, Sydney, New South Wales, Australia; 4 Richard Bright Renal Unit, North Bristol NHS Trust, Westbury on Trym, UK; 5 Lay Advisory Group, Kidney Research, Peterborough, UK

**Keywords:** Chronic renal failure, End stage renal failure, Dialysis, Decision Making, Aged, 80 and over, Health Literacy

## Abstract

**Abstract:**

**Objectives:**

Older people with kidney failure often have a limited range of treatment options, with few being well enough to receive a transplant. Instead, they either start dialysis or have ‘conservative kidney management’ (CKM). CKM involves care that focuses on managing the symptoms of kidney failure and maintaining quality of life in the absence of dialysis. The relative ability of dialysis and CKM to make older people live longer and feel better is uncertain. This study aimed to describe how older patients understand and decide between dialysis and CKM, as evidence suggests they may not be fully supported to make informed decisions between these treatments.

**Design:**

Qualitative study using semistructured interviews, analysed using inductive thematic analysis and constant comparative techniques.

**Setting:**

Three UK specialist kidney units.

**Participants:**

Adults with estimated glomerular filtration rate (eGFR) <15 and aged over 80 years, irrespective of comorbidity or over 65 if living with two additional long-term conditions or frailty. Participants were purposively sampled to maximise clinicodemographic variation, and recruitment was continued until no new major themes were arising in the analysis.

**Results:**

Eight men and seven women with a median age of 81 (range 65–90), and a median eGFR of 12 were interviewed. Three themes were identified: (1) ‘Do dialysis or die’, where not having dialysis was equated with death; (2) The ‘need’ for dialysis, where haemodialysis was perceived as the default treatment and (3) Weighing-up quality and quantity of life, relating to the trade-offs made between treatment benefits and burdens. Participants appeared unlikely to recognise the uncertain survival benefits of dialysis.

Our study took place in England and all the participants were white British. As culture and faith can play a large part in decisions involving life and death, our findings may not be applicable to those in other communities. Participants were recruited from three centres, limiting the breadth of approaches to kidney failure management.

**Conclusions:**

For older people who face short lives irrespective of treatment for kidney failure, unfamiliarity with treatment options, the desire to live and the ‘do or die’ notion conspire to cast haemodialysis as inevitable, regardless of whether this is the most appropriate treatment. To best enable shared decision-making, clinicians should present kidney failure treatment options in an accurate and balanced way, and respect and support older people who are deciding whether to have CKM or dialysis. This includes articulating uncertainty and supporting patients to make trade-offs in relation to what is important to them.

**STRENGTHS AND LIMITATIONS OF THIS STUDY**

- Rigorous qualitative methods.
- Inclusion of older people who had not started kidney replacement therapy.
- Participants sampled from just three centres.
- All participants were white British.

## Introduction

 The highest incidence of kidney failure is seen among people aged 65 years and over,^1^ and current services are likely to be overwhelmed with increased demand in the near future.^2^ Guidelines advocate treatment planning for those at risk of kidney failure,^3^ including shared decision-making between treatment options. The presence of two or more long-term health problems is the norm for older people living with kidney failure,^4^ and the majority experience frailty.^5^ The impact of medication management, medical visits, laboratory tests, lifestyle changes and monitoring can easily exceed individuals’ capacity to cope.^6^ Only 1% of over 75 years with kidney failure receive transplants.^7^ This means that most older people who start dialysis will continue it until they die. The majority start in-centre haemodialysis (HD),^1^ despite evidence that this may be the most intrusive option^8^ and the availability of peritoneal dialysis (PD), provided at home. Conservative kidney management (CKM) describes care focused on managing the symptoms of kidney failure and maintaining quality of life in the absence of dialysis. The comparative survival and quality of life benefits of dialysis and CKM remain unclear but appear to be diminished as people age and develop frailty and additional health conditions.^9^ A systematic review showed survival among older people with kidney failure (of median age 77 years) was 73% at 1 year in those treated with dialysis, and 71% in those receiving CKM.^10^ At 2 years, survival was 62% for those receiving dialysis and 44% for CKM.

The guiding principle of shared decision-making is to align treatments with a patient’s preferences, goals and prognosis. However, there appears to be variability and flaws in decision support for people approaching kidney failure. These include approaches to care that favour HD over other treatments; poorly timed and inadequate information; unfavourable power dynamics between patients and clinicians and insufficient consideration and support for emotional aspects and impact.^11 12^ While there are data examining treatment decision-making for older people with kidney failure, including from the UK,^13^ only a handful of studies have examined decisions between dialysis and CKM from the perspective of those yet to start treatment.^14^,^19^ These studies indicate that older people facing kidney failure experience low awareness and understanding of CKM,^17 18^ inadequate accounting for values and goals,^18^ and that some patients feel they have no choice but to pursue dialysis.^14^ Little work has looked at how older people with kidney failure comprehend and interpret the unclear comparative benefits and burdens of dialysis, which have become clearer over the last decade,^9^ and how people factor in their understanding and expectations of available treatments when deciding which to pursue. This qualitative study was developed to update and obtain a more in-depth understanding of the choices made between dialysis and CKM than is available from the existing literature—exclusively considering older people with kidney failure who have not started kidney replacement therapy (KRT). These data were collected as part of a programme of work exploring preferences for kidney failure treatments.^20^ The findings are expected to inform how to better support older people living with kidney disease, ensuring treatment choices fit with what is important to them.^21^

## Methods

### Design

The presented analysis represents the qualitative component of an exploratory sequential mixed-methods^22^ study, examining the treatment preferences of older patients deciding between dialysis and CKM. Semistructured interviews were used to examine patients’ perceptions of the treatment options for kidney failure and how decisions between these options were made. The findings were used to design a quantitative study (a discrete choice experiment) published separately.^20^ Reporting is in accordance with the Consolidated Criteria for Reporting Qualitative research (see online supplemental materials).^23^

### Participants

English-speaking patients receiving specialist CKD care were eligible if they had an estimated glomerular filtration rate (eGFR) <15 mL/min/1.73 m^2^, were aged over 80 years irrespective of comorbidity or were aged over 65 years if they had a Davies comorbidity score ≥2^24^ or a WHO performance status score of ≥3.^25^,^27^ Individuals were excluded if they had ever received outpatient dialysis or a transplant. Patients were recruited from three hospitals situated between the North and Southwest of England: two transplanting centres each providing care to approximately 600 dialysis recipients and a non-transplanting centre with approximately 200 dialysis recipients. Both larger centres provided subspecialist CKM multidisciplinary care, while the smaller centre provided CKM within general nephrology services.

### Data collection

Patients were purposively sampled from general nephrology clinics in the main and peripheral kidney units of the three hospitals to maximise variation in age, sex, ethnicity, clinically documented treatment plan and socioeconomic background. Local nephrology teams (doctors and nurses reviewing the patients) assessed eligibility and informed potential participants of the study either by telephone or at the time of a routine hospital visit. Potential participants were provided with an information leaflet and invitation letter. Clinical teams emailed the research team with the contact details of people who expressed willingness to take part. Unless these potential participants called ahead or returned the provided slip to decline, BH telephoned them to organise interviews. Written consent, planned treatment and sociodemographic information (age, gender, ethnicity, years of full-time education, WHO performance status and occupation) were collected at the time of interviews. One interview was conducted with each participant between September 2018 and July 2019 in patients’ homes by BH, a white, male, trainee kidney specialist in his late 30s. This was BH’s first experience of qualitative research, conducted as part of his PhD, which included formal training in qualitative research and interviewing skills. No other people were present during interviews. Clinical teams provided patients’ clinically documented treatment plan, latest eGFR, list of comorbid conditions and cause of kidney failure. An Index of Multiple Deprivation was calculated using participants’ postcodes.^28^

An initial topic guide was developed using the literature and piloted with patient input. Following initial analysis, an enhanced topic guide was used in the second and third hospital sites, which was adapted during the concurrent analysis process to enable further exploration of initial themes and patterns in the data (online supplemental file). Transcript review and interview coaching were provided by JC, LS, RM and LR. Unless directly asked (this happened once), BH did not disclose his medical training and described himself as a ‘researcher’. Interviews were audio recorded using an encrypted digital voice recorder, and handwritten field notes taken. Interviewees received £20 vouchers to compensate for their time. Recruitment was continued until no new major themes were arising in the analysis, at which point sufficient information power^29^ was considered to be available to support the findings. Participants were not sent their transcripts nor involved in analysis.

### Data analysis

Recordings were transcribed verbatim and managed with QSR NVivo V.11 software.^30^ Transcripts were analysed inductively, using thematic analysis^31^ and constant comparative techniques, originating in grounded theory.^31^ Initial coding was completed by BH. Starting with ‘open’ coding, concepts and meanings within interviews were identified from patients’ views and experiences. The first three interview transcripts were line-by-line coded and discussed at face-to-face researcher meetings before the fourth interview was conducted. Codes and interviews were discussed and compared, with abstract consideration of wider meaning, alongside reorganisation and recoding, and thematic development.^32^ LS and LR subsequently coded two interviews each, and a selection of transcripts were also read independently by JC, FC and RM and discussed as a team to refine the coding framework and interpretation. BH wrote three in-depth descriptive accounts on subsets of interviews, which were shared and discussed at alternate-monthly research meetings and ultimately formed into a final analytical account. Seeking negative cases (those that appear to contradict explanations in the data) was part of the purposive recruitment strategy and constant comparative approach. Negative cases were used to explicate initial analytical findings, add richness to the analysis and generate further thematic exploration. Analysis and recruitment were conducted in parallel and discontinued when no new themes were identified.^33^

### Patient and public involvement

A panel of people with lived experience of kidney failure and their family members were involved from inception in study design and oversight, including development of patient-facing materials, the interview topic guide and data interpretation. MS coauthored the manuscript.

## Results

### Participants

33 individuals were approached to take part, of whom 15 (45%) were interviewed. Of the 18 (55%) who did not take part, 7 returned paper slips declining participation, and 4 had a family member call to decline. Reasons offered for non-participation included being too busy (2), memory problems (1), deafness (2), being away (1), having started dialysis (1) and being in hospital (2). The remaining individuals did not offer a reason.

Interviews lasted a median of 63 min (range 29–84). Participant characteristics are presented in table 1. Eight men and seven women took part, with a median age of 81 years (range 65–90), and a median eGFR of 12. 10 participants had diabetes mellitus as a cause of kidney disease, 3 had vascular/hypertensive disease and 2 had nephrectomy for cancer. All described their ethnicity as white British. Clinically documented treatment plans were available for each participant, with seven preparing for in-centre HD, two for PD and the remaining six for CKM. No participants were active on the transplant waiting list. Two participants voiced uncertainty about their clinically documented plan, one of whom was considering CKM instead of HD another PD instead of CKM.

**Table 1:** Participant characteristics (n=15)

Participant characteristic (n=15)	Number (%)*
Gender	
Female	7 (47)
Male	8 (53)
Age	
65–69	2 (13)
70–74	1 (7)
75–79	2 (13)
80–84	5 (33)
85–89	4 (27)
≥90	1 (7)
eGFR (mL/min/1.73 m^2^)	
<10	4 (27)
10–14	10 (67)
≥15	1 (7)†
Treatment plan	
HD	7 (47)
PD	2 (13)
CKM	6 (40)
Active on transplant waiting list	0 (0)
Major comorbidities	
Type 2 diabetes	10 (67)
Ischaemic heart disease	7 (47)
Hypertension	5 (33)
Malignancy	4 (27)
Obesity	2 (13)
Heart failure	1 (7)
Stroke	1 (7)
Other comorbidity	5 (33)
Cause of kidney disease	
Type 2 diabetes	10 (67)
Hypertension and/or vascular disease	3 (20)
Removal of kidney cancer	2 (13)
WHO performance status	
0	0 (0)
1	5 (33)
2	2 (13)
3	8 (53)
4	0 (0)
Years of full-time education	
0–5	1 (7)
6–10	6 (40)
11–15	6 (40)
16–20	2 (13)
IMD	
1–2	4 (27)
3–4	3 (20)
5–6	6 (40)
7–8	1 (7)
9–10	1 (7)

* Due to rounding, percentages may not always appear to add up to 100%.

† One participant who had an eGFR of 25 mL/min/1.73 m2 at the time of interview, having been 14 mL/min/1.73 m2 at recruitment.

CKM, conservative kidney management; eGFR, estimated glomerular filtration rate; HD, haemodialysis; IMD, Index of Multiple Deprivation; PD, peritoneal dialysis.

Illustrative quotes are provided in italics, including divergent views and negative cases, where relevant. All participants were assigned a pseudonym. Quotes are marked with the participant’s pseudonym, age and clinically documented treatment plan in the following format: (name; age in years; abbreviated treatment plan: haemodialysis—HD, peritoneal dialysis—PD, conservative kidney management—CKM). For example, Alice, an 85 year old woman planning for peritoneal dialysis: (Alice;80s;PD).

### Findings

Participants described how they prepared for kidney failure in the face of a life-changing diagnosis and an unpredictable future. For most, recognition that kidney failure was impending appeared to have been seminal, transforming a minimally intrusive disease into one influencing life and death. Many recalled intensely negative experiences, typically triggered during consultations where treatments for kidney failure were first discussed. Those who were diagnosed late in the disease course, for example, Jeremy, who learned of his kidney disease when his eGFR was in the low 20s, described especially intense feelings of ‘*shock’*:

My first thoughts about this thing were absolute shock. Despair really. (Jeremy;80s;HD)

However, even participants who had years of preceding chronic kidney disease monitoring, for example, Betty, who had type two diabetes mellitus, and understood that her ‘*kidneys were at risk’*, expressed surprise when the prospect of kidney failure was raised. For some, this appeared to reflect the fact, or implications, of declining kidney function had not been successfully communicated. However, accounts also suggested that kidney failure was understood as a separate, more severe condition, rather than an advanced stage of chronic kidney disease. This appeared bound up with the concept that kidney failure without dialysis was akin to death, establishing a ‘do or die’ paradigm (theme 1): the perspective that dialysis must be initiated, or life would end. Related to this was a depiction of dialysis as ‘needed’ (theme 2), reflecting the consequence of ‘do or die’, alongside unfamiliarity with CKM as a treatment option, and apparent norms framing dialysis as the default treatment. Meanwhile, participants almost universally discussed the inevitability of their death and anticipated burdens from dialysis. They appeared to intuitively ‘weigh up’ (theme 3) the quality and quantity of life consequences of futures with and without dialysis.

### Theme 1: ‘do dialysis or die’

Individuals did not typically consider their treatment plan to reflect a decision from a set of options, including CKM. Rather, initiation of kidney replacement therapy was depicted as life-sustaining, and a decision to decline dialysis was depicted as turning down the longer life dialysis would bring. This view was clearest among individuals anticipating dialysis initiation, who largely depicted negligible life expectancy without dialysis. Three participants—all preparing for dialysis—framed a decision to decline dialysis as actively shortening life, akin to suicide or euthanasia: “*letting somebody else kill you*” (Jeremy;80s;HD). For some, declining dialysis appeared to reflect the acceptance of death from kidney failure:

I knew that doing nothing, I would become progressively worse so—Shorten your life in other words. (Derrick;80s;HD)

Many participants appeared to consider prognosis as unpredictable. Some described this in terms of risk or fate, “*It’s just as long as it is*” (David;60s;HD). Others appeared to make prognostic estimates influenced by their age, comorbidities and life experiences. Participants’ expectations varied greatly—ranging between those who felt they were at the very end of life, “*I’m on my last legs*” (Sally;80s;CKM), and others anticipating decades: “*if it’s 40 years, it’s ok*” (David;60s;HD). Some participants appeared to expect that dialysis would return life expectancy to what it would have been without kidney disease. For these individuals, the perceived survival benefit of dialysis appeared to revolve around how long they expected to live in the *absence* of kidney failure:

Well, I don’t know. I mean something else could happen. I could have a heart attack. You don’t know what your end’s going to be. (Brian;70s;PD)

The accounts of participants planning for dialysis did not tend to include speculation about the effects of their choice on the kind of end-of-life experiences that might ensue, and the concept of dialysis discontinuation appeared universally unfamiliar.

[Interviewer] Have you ever thought about whether people stop dialysis having started it?

[Participant] No I’ve never heard of anybody not doing it. (Jeremy;80s;HD)

Seven participants—including five of the six preparing for CKM—made less stark survival comparisons when comparing futures with and without dialysis. Some reported that the additional benefit to survival from dialysis initiation may be slight, given their age or other illnesses. Some individuals simultaneously held the ‘do or die’ paradigm and the concept that dialysis may not extend their life by long:

It’s basically ‘have that or die’… [but] if you do have the dialysis what are they going to gain me, an extra six months, or a year? (Joe;70s;HD)

In summary, this first theme captured how participants depicted that they were offered dialysis, perceived as a treatment to prolong life. The magnitude and framing of this potential survival benefit appeared to be associated with participants’ willingness to accept that they could choose not to pursue dialysis.

### Theme 2: the ‘need’ for dialysis

Initiation of kidney replacement therapy following kidney failure often appeared as a fait accompli. Many participants recalled having been told years or decades before that they would eventually require dialysis, with initiation widely referred to in depictions of certain futures, including directive terms, such as ‘having to’ or ‘needing to’ start dialysis. Treatment for kidney failure often appeared to be synonymous with in-centre HD, and familiarity with, knowledge and understanding of this (often referred to simply as ‘dialysis’) appeared to surpass that of PD, CKM and transplantation. Indeed, the fact that there were alternatives to HD appeared to have come as a surprise to several participants who had been visiting the kidney clinic for many years:

From the start then I knew that in twenty years I’d probably be on dialysis… I didn’t know the second, third options [peritoneal dialysis and CKM] were there. I assumed on dialysis. (Brian;70s;PD)

All other participants receiving or expecting to receive CKM recognised dialysis as having been an option, but portrayed themselves as having declined dialysis, rather than as having made an active choice to pursue CKM. Even those expecting CKM often appeared to have limited understanding of what it would involve, depicting a ‘status quo’ option, rather than the introduction of a new treatment or framework for care provision:

You might as well go the normal route [die without starting dialysis] and take what’s coming to you. (Betty;80s;CKM)

Where the concept of CKM was discussed by those preparing for dialysis, it was typically presented as a ‘do nothing’ option:

We were talking dialysis, and to see what all the options are, I said “what if I don’t do anything about it, you know?” (Derrick;80s;HD)

A minority of participants recalled being informed that one or more potential treatments were impossible for them, with some recalling how they had been restricted to just one option. Transplantation was widely perceived as unattainable, though few recalled being informed of this by their clinical team. Three participants under the age of 80 described themselves as awaiting review of their eligibility for transplantation. For those who perceived themselves as ineligible, age was widely advanced as the reason:

They started talking about “have you thought about what your treatment’s going to be eventually” and I said “well, I suppose having a transplant possibly”. “Oh no, no, no” he said, “too late for that, too late for that, at your age”, he said, “I wouldn’t recommend a transplant, you know, you’ll have to go on dialysis”. (Brian;70s;PD)

A minority of participants alluded to the idea that their future treatment remained undecided or could change. For some, this appeared to reflect an understanding that future declining health might influence their attitude towards dialysis. Other individuals preparing for dialysis discussed temporising or avoiding the decision to prepare for dialysis, but presented this as compatible with its inevitable initiation:

If it comes to it, I might have it at home or I might even not bother, because I’m not as good as I was. (Beryl;80s;CKM)

I know the dialysis is going to come, but I don’t want to think about it, you know? (David;60s;HD)

In summary, this second theme captured how many participants appeared to conceive of dialysis as an inevitability, unless they declined initiation or died from a competing cause before reaching the putative dialysis initiation point.

### Theme 3: weighing-up quality and quantity of life

A ‘weighing up’ of pros and cons was universal, where individuals described selecting their treatment from several options. The assumed extension to life provided by dialysis needed to be of acceptable quality, and participants’ capability to live and undertake activities independently appeared to be critically important:

If it’s going to give me a reasonable quality of life, then it will keep me going. If I didn’t think that I would have a reasonable quality of life, then I would take the option of nothing. (Muriel;60s;HD)

Participants appeared to consider the routine of dialysis as unpleasant. Intrusion into daily life was consistently cited as negative, and some participants were concerned life would become ‘centred around’ (Brian;70s;PD) treatment. The time used for dialysis was frequently portrayed as ‘wasted’ (multiple participants). Few anticipated feeling better after initiation:

You have to get transported to hospital and back again. So, you can imagine you leave home about eight o’clock in the morning and you’d be lucky if you got home at six o’clock at night. Well, that would be fun, wouldn’t it? (laughs) (Betty;80s;CKM)

Only one participant suggested a positive aspect of dialysis beyond its influence on survival and symptom control—anticipating social interaction as part of treatment:

If I go to the hospital, if nothing else there’s going to be a nurse or a tea lady to have a chat with. Company. (Muriel;60s;HD)

Some appeared to consider the orchestration of home dialysis and associated equipment as an intrusion that they were not willing to accept. For those expecting to start dialysis, the negative aspects tended to be framed as justified:

I know it’s a drag going to hospital three times a week, but at least I’m here to do it. (Derrick;80s;HD)

Older, frailer and largely unpartnered participants spoke of a decline in their ability to partake in pleasurable activities and adaptation to changes in capability. The concept of a complete or ‘good life’ (Clive;80s;CKM) was pervasive. However, rather than considering their current life ‘not worth living’ (Derrick;80s;HD), for participants who were preparing for CKM, it was a putative future life on dialysis that was considered unacceptable. For this group, the negative aspects of dialysis were framed as dominant, even where a longer life was anticipated, were they to start it:

Ok so you’re going to have a longer life, but what life is it? (Clive;80s;CKM)

For some, the trade-offs between their anticipated future on dialysis and one without appeared closely balanced. This seemed to fuel uncertainty about whether their planned treatment was right for them, or an expectation that they might not undertake their clinically documented plan:

I do have great reservations as to whether any of it’s needed and whether it’s actually worth the while? This is only a temporary respite and that you’re going to die anyway… All seems quite a horrible process, and as I say, I think it’s a bit of a last-ditch thing, you know, to keep you running for a little bit longer. (Joe;70s;HD)

In summary, this third theme captured how all participants appeared to weigh up the positive and negative aspects of futures with and without dialysis, allowing them to evaluate their anticipated treatment and compare this with alternatives.

## Discussion

This UK study looked at treatment decisions between dialysis and CKM, exclusively among older people with kidney failure who have not started KRT. We examined how individuals comprehended and interpreted the comparative benefits and burdens of treatments.^9^ We found that treatment plans were made in the context of participants having already accepted the serious nature of their condition and the possibility of death as a result. Few participants—irrespective of their age or levels of comorbidity—appeared familiar with the uncertain survival benefits of dialysis. For many, a future without dialysis did not appear to be perceived as a real option; replaced with a ‘do or die’ Hobson’s choice.^14^ Those opting for dialysis did not appear to have been fully supported to consider the implications of their decision on their remaining lives, including where or how they might die. Unfamiliarity with home therapies and CKM appeared to render HD the default treatment that would eventually be ‘needed’ for life to continue. Meanwhile, participants considered the life they expected to live when appraising treatments and readily made trade-offs between their benefits and burdens.

To decline dialysis appeared to be a viable option only to those who perceived that the presumed survival benefit might be outweighed by the burdens of treatment. Choosing CKM appeared to involve going against the grain—‘opting-out’ from dialysis.^34^,^39^ In keeping with the literature,^15 16 34 35 40 41^ there was no evidence that our participants actively opted for palliative care. Rather, they rejected a future life on dialysis.^42^ Critically, this did not indicate that they considered their current life intolerable.

It has been shown in patients of all ages that kidney failure treatment preferences reflect trade-offs^43^ between anticipated benefits—principally survival on dialysis^15 16 44^ and the influence on independence, daily life, responsibilities and interests.^11 12 19 41 45^ The trade-offs that older people make are likely to differ from those made by younger, potentially transplantable people living different occupational, social and familial lives.^11^ Longevity is rarely paramount for people with life-limiting illness, who value support for themselves and loved ones, and prioritise independence, meaning, comfort and achievement of life goals.^46 47^ While kidney replacement therapy is plainly life-prolonging for those whose survival is dominated by their kidney disease, the comparative survival and quality of life benefits of dialysis and CKM remain unclear for many older people.^9^ Some who initiate dialysis will die close to—or even before—the point that they would have died, had they never started. Those at the highest risk of competing mortality—the oldest and those living with major comorbidities—are most likely to prepare for or receive treatment that does not prolong their lives.^9^

Conflation between CKM and ‘no treatment’^1317 18 48^,^51^ or death^14 16 39^ may undermine individuals’ freedom to make trade-offs between the uncertain comparative benefits and burdens of dialysis and CKM. That a ‘non-choice’ can arise from the misperception of less invasive care as ‘doing nothing’ has long been recognised in cancer^52^ where patients can be steered towards anticancer treatment, irrespective of likely treatment benefit.^53^ Meanwhile, which treatments individuals prepare for and initiate profoundly influences their experience of living and dying. For example, those who choose dialysis appear more likely to be hospitalised and to die in hospital than others who opt for CKM.^9^ Exaggerated impressions of the survival implications between dialysis and CKM are likely to lead some to prepare for dialysis, despite CKM being a better fit for their preferences to minimise treatment intrusion.

It has been shown before that people with kidney disease may be left to deduce which treatment options are available to them^11 34^ and are not always provided with the information or support needed to ensure their treatments fit their preferences.^54 55^ Our study portrays a one-dimensional system of decision-making, where the trade-offs bound up in a potentially longer life with dialysis, and a potentially shorter life without, did not appear to have been successfully facilitated. It may appear that little progress has been made since earlier studies suggested deficiencies in decisional support.^13^ This raises the question as to whether clinicians believe and feel able to convey the uncertain survival benefits of dialysis and highlights the need to develop ways of helping patients to weigh up the benefits and burdens of treatments.

Older people facing the prospect of kidney failure are likely to benefit from tailored approaches to decision-support. This must reflect where they are in the life course and what a future with kidney failure might look like for them. The three themes identified in this study provide clues as to how their care might be adapted. Clinicians will need training and resources to successfully convey uncertainty, support the weighing-up process around factors of importance to the individual, and challenge the idea that dialysis is the default. Consistency across clinical teams and over time is challenging,^56^ and CKM services must be available and sufficient.^13^ How CKM is depicted and conceived appears central. Framing CKM more accurately can improve patients’ perceptions.^17^ Driven by the perceived need to offer ‘positive alternatives to dialysis’ (Davison *et al*^57^ pg.453), efforts have been made to define and standardise CKM.^58^ This is important, given that access to and models of kidney supportive care are inconsistent^59 60^—meaning that in some places, a choice to not pursue dialysis does not lead to receipt of CKM. However, fully establishing CKM as a viable alternative to dialysis may require patients and clinicians to be persuaded that the ‘do or die’ paradigm is a fallacy born from envisaging dialysis as ‘needed’ to prevent death. If this were to be true, CKM could never be received, since those who ‘need’ but don’t start dialysis would just die. While patients and clinicians may perceive that the choice is between dialysis and death, this is not the decision being made. Median survival from treatment decision-making or reaching kidney failure ranges between 20 and 67 months for dialysis and 6 to 31 months with CKM, depending on age and other factors.^61^ Individuals may need to be helped to understand that the absolute survival advantage of dialysis can be small, given their shorter prognosis. This will require prognostic honesty, perhaps with the sharing of absolute survival estimates and is most likely to be successful if decision-support involves routine discussion and documentation of individuals’ goals for treatment of kidney failure. Living longer is rarely the sole determinant of treatment choice.^20^ Individuals who are supported to contextualise the reasons for either dialysis initiation or for choosing CKM will be better placed to make decisions based on their preferences.

The strengths of this study include a rigorous application of qualitative methods and a broad range of clinicodemographic variation between study participants, sampled from multiple kidney centres. Including individuals who had not started kidney failure treatment ensured the findings were relevant to those making preparatory decisions. The study has limitations. Participants were recruited from three centres, limiting the breadth of approaches to kidney failure management. The frequency and approach to CKM differ between kidney units,^13 60 62^ which may influence the transferability of findings. The sample size is small, though this reflects the fact that sufficient information power arose early to support the major themes. Despite efforts to recruit from diverse ethnic backgrounds, all participants were white British. Culture and faith play important roles in understanding disease and treatment decision-making,^63 64^ so our findings may not be typical for members of other communities, and further research with ethnically diverse groups is needed. Interview studies can only capture participants’ accounts of clinical encounters. These encounters appear critically important in forming people’s perceptions of their treatment options, and observation and analysis of clinician–patient interactions may help to uncover which consultation approaches work best.^65^

In conclusion, this study identified that an assumption that life will end unless dialysis is started, alongside unfamiliarity with and misperceptions regarding treatment options conspire to cast HD as the default treatment for kidney failure. The influence kidney specialists have on patients’ understanding and expectations of care means they must be trained to ensure patients can make shared, informed decisions. Clinicians must support patients to make trade-offs between the uncertain benefits and requisite burdens of dialysis and CKM. Better evidence will help. Meanwhile, redefining the ‘need’ as the ‘reason’ for dialysis initiation, and reframing the ‘do or die’ fallacy by sharing absolute survival predictions with dialysis and CKM might facilitate improvements in person-centred decision-making.

## Supplementary material

10.1136/bmjopen-2024-095185 online supplemental file 1

10.1136/bmjopen-2024-095185 online supplemental file 2

## Data Availability

Data are available on reasonable request.
