# Supplementary material for: ‘It’s basically ‘have that or die’’: a qualitative study of older patients’ choices between dialysis and conservative kidney management
Source: BMJ Open. 2025 Mar 7;15(3):e095185. doi: 10.1136/bmjopen-2024-095185 (PMC11891532; doi:10.1136/bmjopen-2024-095185)
Supplement: online supplemental file 1 [file bmjopen-15-3-s001.pdf]

The UNPACK Study – Interview Topic Guide, Phase 1 Qualitative Interviews (patient, family member or both)

- Check they received **UNPACK Phase 1 Interviews, Patient information sheet**
- Explain interview and duration, including audio-recording
- Negotiate whether interview will be individual, or two-person (patient/family member)
- Confirm that patient is happy for their healthcare to be discussed with family member
- Obtain informed consent
    - Capacity assessment:
        - Ask whether they would like to continue to an interview
        - Ask what they understand would happen if they declined an interview
        - Reiterate that this decision will not impact care
        - Check for further questions
    - Complete **UNPACK Phase 1 Qualitative Interviews, Consent Form** ☐

Interview - Start audio recorder [date of recording _____________]

- *"The point of these interviews is to help me to understand what is important to people when thinking about treatments for kidney disease. How do these 'important things' influence what treatments are chosen? Who and what else is involved?*
- *There are no right or wrong answers – I'm interested in your experiences, thoughts and ideas. Whatever you say will be useful to me – I'm here to learn from you.*
- *You can stop the interview or change the subject at any point – please just say."*

Tell me a little about yourself…

- *Still working/used to work as?*
- *How so you spend your time?*
- *Hobbies/interests?*

When kidney problems first diagnosed?

- *What happened?*
- *Since?*
- *Now?*
- *Expectations?*

What discussions about future have happened?

- *With whom?*
    - *Kidney team*
    - *Family/friends*
    - *GP*
    - *Other patients?*
- *What discussed?*
- *Why were they had?*
- *Were decisions made?*
    - *How?*
    - *Who?*

What (RRT) treatments have been discussed?

- *Initial feelings about these?*
- *Feelings about them now?*
- *Understanding of what is involved?*
- *Who and how did you learn about them?*
- *Likelihood of needing?*
- *Transplantation?*
- *Thoughts about receiving dialysis?*
    - *Effects on self*
    - *Others*
- *Thoughts about receiving conservative care?*
    - *Self*
    - *Others*

What do you think might be the advantages/ disadvantages of planning now, vs. waiting to see?

Do you feel like you have a plan?

- *If not, why not, would you want one?*
- *If so, how/when?*
- *Process: gradual/one off?*
- *Did you feel like there were alternatives?*
- *Who there? Who chose?*
- *Is this how you usually make decisions?*

Have you thought about end of life care?

- *Have you talked? Who? How?*
- *How might these plans influence?*

Have you thought about loss of capacity?

- *What plans made?*
- *Lasting power of attorney?*

- *Would you like to receive updates about this research?* ☐
- *Copy of transcript?* ☐
- *Check I have all Information I need… age* ☐ *sex* ☐ *ethnicity* ☐ *marital status* ☐ *who you live with* ☐
- **Thanks. <u>Stop and pack</u> audio recorder.**
