# Supplementary material for: ‘It’s basically ‘have that or die’’: a qualitative study of older patients’ choices between dialysis and conservative kidney management
Source: BMJ Open. 2025 Mar 7;15(3):e095185. doi: 10.1136/bmjopen-2024-095185 (PMC11891532; doi:10.1136/bmjopen-2024-095185)
Supplement: Supplementary file 3 [file bmjopen-2024-095185.reviewer_comments.pdf]

PEER REVIEW HISTORY

BMJ Open publishes all reviews undertaken for accepted manuscripts. Reviewers are asked to complete a checklist review form and are provided with free text boxes to elaborate on their assessment. These free text comments are reproduced below.

ARTICLE DETAILS

Title (Provisional)

“It’s basically ‘have that, or die’” – a qualitative study of UK older patients’ choices between dialysis and conservative kidney management.

Authors

Hole, Barnaby; Rooshenas, Leila; Morton, Rachael; Caskey, Fergus; Scanlon, Miranda; Coast, Joanna; Selman, Lucy

---

VERSION 1 - REVIEW

**Reviewer**	**1**
**Name**	**Karlin, Jennifer**
**Affiliation**	**UC Davis, Family and Community Medicine**
**Date**	**11-Nov-2024**
**COI**	**None**

Thank you for the opportunity to review this paper. It is very clear and well written. I have minor suggestions:

1) Intro- excellent

2) Methods- under data collection, can you please describe where you sampled from? Which clinics? Which locations, etc.

3) Methods- what were the clinical teams like? Who were they? Nephrology teams? Internal medicine?

4) your descriptions of the research team are great, as is the description of development of the interview guide (with patient input!)

5) The results have a great balance of qualitative examples. Themes are well laid out.

6) The discussion is also strong. However, I think that you might improve it by adding in context from other illnesses. It would be helpful to hear what the authors think is similar or not to cancer diagnosis and treatment options, for example.

Once these minor issues are addressed, I think this will be a great addition to the literature.

**Reviewer**	**2**
**Name**	**Finderup, Jeanette**
**Affiliation**	**Aarhus Universitetshospital, Renal Medicine**
**Date**	**17-Nov-2024**
**COI**	**None**

Thank you for interesting study investigating an important topic. Some suggestions for improvement of the study:

Title

An abbreviation in the title and the running head is not nice although it is UK. UK is not important in the heading at all.

Abstract

'Kidney failure is most common amongst older people', is the right and what does it mean. Something is missing. What do you compare to? Later on in the introduction it is clear, that you talk about the highest incidence, but that is not clear in the abstract.

You state that kidney replacement therapy aims to prolong life and manage systems, but I think, that is also what CKM aims to. You state that CKM aims to optimize quality of life, but I think, that is also what dialysis aims to. So your definition of dialysis and CKM and their distinction is lacking. You describe CKM and dialysis in another way in the plan-language summary, e.g CKM… …on managing the symptoms.

Introduction

When you decide between dialysis and CKM, then transplantation is not a relevant option. Instead of you should elaborate the different dialysis modality, for again, home haemodialysis is not a relevant option for people choosing between dialysis and CKM. But assisted PD is a relevant option. Later on, you elaborate, but this sentence does not work: including shared

decision-making between peritoneal dialysis and haemodialysis, conservative kidney

management (CKM), and transplantation. To state in the introduction that dialysis is a destination, makes it not clear, that other options exist.

Results

Only two participants have chosen PD.

'All other participants receiving or expecting to receive CKM recognised dialysis as having

been an option, but portrayed themselves as having declined it, rather than as having made

an active choice to pursue palliative care.', why do you suddenly talk about palliative care and not CKM.

You label some participants younger, but do not define it further.

You have chosen a very huge variation in age. I think the decision for people below 75 years is more about choosing between different dialysis modality and the decision between dialysis and CKM is more relevant for people above 75 or 80 years. I think it is a problem that you include so young participants. You need to make this clearer in the findings, which findings are only related to the older group and which are only related to the younger group or maybe you should exclude the younger group.

Discussion

You state that you are the first to look at this type of decision in a UK population. This is not true, both Nicola Thomas and Anna Winterbottom have looked into this type of decision.

Again you use the term 'palliative care', but it is not clear how you define this term and distinct the term from CKM.

Supplementary material

The interview guide include a comment from a co-worker

VERSION 1 - AUTHOR RESPONSE

Reviewer: 1 Dr. Jennifer Karlin	
Intro- excellent/ your descriptions of the research team are great, as is the description of development of the interview guide (with patient input!)/ The results have a great balance of qualitative examples. Themes are well laid out/ The discussion is also strong. Once these minor issues are addressed, I think this will be a great addition to the literature.	We thank Dr Karlin for her positive feedback, and for the pointers towards improving the manuscript.
Methods- under data collection, can you please describe where you sampled from? Which clinics? Which locations, etc.	We have rephrased:<br>*"Patients were purposively sampled from general nephrology clinics in the main and peripheral kidney units of the three hospitals to maximise variation in age, sex, ethnicity, clinically documented treatment plan and socioeconomic background."*<br>[1st para, data collection, pg6]
Methods- what were the clinical teams like? Who were they? Nephrology teams? Internal medicine?	We have rephrased:<br>*"Local nephrology teams (doctors and nurses reviewing the patients) assessed eligibility and informed potential participants of the study either by*

	telephone, or at the time of a routine hospital visit.”<br>[1st para, data collection, pg6]
Discussion- I think that you might improve it by adding in context from other illnesses. It would be helpful to hear what the authors think is similar or not to cancer diagnosis and treatment options, for example.	Thank you – this is an area that we are keen to explore further. We have added a few contextualising comments:<br><br>*“Longevity is rarely paramount for people with life limiting illness, who value support for themselves and loved ones, and prioritise independence, meaning, comfort, and achievement of life goals.”*<br><br>and<br><br>*“That a ‘non-choice’ can arise from the misperception of less invasive care as ‘doing nothing’ has long been recognised in cancer where patients can be steered towards anticancer treatment, irrespective of likely treatment benefit.”*<br>[Discussion page 15]
Reviewer: 2 Dr. Jeanette Finderup	
Thank you for interesting study investigating an important topic. Some suggestions for improvement of the study.	We thank Dr Finderup for her feedback and suggestions for improvement.
Title- An abbreviation in the title and the running head is not nice although it is UK. UK is not important in the heading at all.	We have removed “UK” from both the main and running titles.
Abstract- 'Kidney failure is most common amongst older people', is the right and what does it mean. Something is missing. What do you compare to?	We have removed this sentence from the abstract.
Abstract- You state that kidney replacement therapy aims to prolong life and manage systems, but I think, that is also what CKM aims to. You state that CKM aims to optimize quality of life, but I think, that is also what dialysis aims to. So your definition of dialysis and CKM and their distinction is lacking. You describe CKM and dialysis in another way in the plan-language summary.	We have replaced this section from the abstract with content from the (removed) plain language summary:<br><br>*“CKM involves care that focuses on managing the symptoms of kidney failure and maintaining quality of life in the absence of dialysis. The relative ability of dialysis and CKM to make older people live longer and feel better is uncertain.”*<br>[Abstract]
Introduction- When you decide between dialysis and CKM, then transplantation is not a relevant option. Instead of you should elaborate the different dialysis modality, for again, home haemodialysis is not a relevant option for people	We have removed our sentence in a rewrite of the first paragraph, attempting to make no assumptions about which treatments are ‘relevant’ to individuals choosing between them:

choosing between dialysis and CKM. But assisted PD is a relevant option. Later on, you elaborate, but this sentence does not work: including shared decision-making between peritoneal dialysis and haemodialysis, conservative kidney management (CKM), and transplantation.	*"Guidelines advocate treatment planning for those at risk of kidney failure, including shared decision-making between treatment options."* [Introduction first paragraph page 4]
Intro- To state in the introduction that dialysis is a destination, makes it not clear, that other options exist.	We have rewritten this sentence as:<br><br>*"This means that most older people who start dialysis will continue it until they die."*<br>[Introduction first paragraph page 4]
Results- You label some participants younger, but do not define it further.	We have rephrased as:<br><br>*"Three participants under the age of 80"*<br>[Results page 11]
Results - 'All other participants receiving or expecting to receive CKM recognised dialysis as having been an option, but portrayed themselves as having declined it, rather than as having made an active choice to pursue palliative care.', why do you suddenly talk about palliative care and not CKM.	We have rephrased the first sentence:<br><br>*"...rather than as having made an active choice to pursue CKM."*<br>[Results page 11]
Discussion- Again you use the term 'palliative care', but it is not clear how you define this term and distinct the term from CKM.	We believe that the term "*palliative care*" is well defined and understood, so have not made a change to its use here – we hope that the preceding amendment clarifies that we are not conflating CKM and palliative care.
Results- You have chosen a very huge variation in age. I think the decision for people below 75 years is more about choosing between different dialysis modality and the decision between dialysis and CKM is more relevant for people above 75 or 80 years. I think it is a problem that you include so young participants. You need to make this clearer in the findings, which findings are only related to the older group and which are only related to the younger group or maybe you should exclude the younger group.	We do not agree with the suggestion that we should exclude our younger participants, who were included because of their comorbidities, and low performance status. The observational literature we cite suggests the survival benefit of dialysis is diminished for them.[1] We have emphasised in the opening of the discussion:<br><br>*"Few participants – irrespective of their age or levels of comorbidity – appeared familiar with the uncertain survival benefits of dialysis"*<br>[Discussion 1st paragraph, pg 14]<br><br>1) Buur LE, Madsen JK, Eidemak I, et al. Does conservative kidney management offer a quantity or quality of life benefit

	compared to dialysis? A systematic review. *BMC Nephrol.* 2021;22(1):307.
Discussion- You state that you are the first to look at this type of decision in a UK population. This is not true, both Nicola Thomas and Anna Winterbottom have looked into this type of decision.	We have removed the claim by rephrasing:<br><br>*"This UK study looked at treatment decisions between dialysis and CKM, exclusively amongst older people with kidney failure who have not started KRT."*<br>[Discussion 1st para pg 14]
Supplementary material - The interview guide include a comment from a co-worker	Thank you – we have removed it.

VERSION 2 - REVIEW

Reviewer 2

Name Finderup, Jeanette

Affiliation Aarhus Universitetshospital, Renal Medicine

Date 22-Dec-2024

COI

Thank you for improving your paper. I do not have further comments.
